# Benchmarking variant identification tools for plant diversity discovery

**DOI:** 10.1186/s12864-019-6057-7

**Published:** 2019-09-09

**Authors:** Xing Wu, Christopher Heffelfinger, Hongyu Zhao, Stephen L. Dellaporta

**Affiliations:** 1 Department of Molecular, Cellular and Developmental Biology, Yale University, New Haven, CT 06520-8104 USA; 2 Department of Biostatistics, Yale School of Public Health, Yale University, New Haven, CT 06520-8034 USA

**Keywords:** Read alignment, Variant calling, Machine learning, Variant filtering, Imputation

## Abstract

**Background:**

The ability to accurately and comprehensively identify genomic variations is critical for plant studies utilizing high-throughput sequencing. Most bioinformatics tools for processing next-generation sequencing data were originally developed and tested in human studies, raising questions as to their efficacy for plant research. A detailed evaluation of the entire variant calling pipeline, including alignment, variant calling, variant filtering, and imputation was performed on different programs using both simulated and real plant genomic datasets.

**Results:**

A comparison of SOAP2, Bowtie2, and BWA-MEM found that BWA-MEM was consistently able to align the most reads with high accuracy, whereas Bowtie2 had the highest overall accuracy. Comparative results of GATK HaplotypCaller versus SAMtools mpileup indicated that the choice of variant caller affected precision and recall differentially depending on the levels of diversity, sequence coverage and genome complexity. A cross-reference experiment of *S. lycopersicum* and *S. pennellii* reference genomes revealed the inadequacy of single reference genome for variant discovery that includes distantly-related plant individuals. Machine-learning-based variant filtering strategy outperformed the traditional hard-cutoff strategy resulting in higher number of true positive variants and fewer false positive variants. A 2-step imputation method, which utilized a set of high-confidence SNPs as the reference panel, showed up to 60% higher accuracy than direct LD-based imputation.

**Conclusions:**

Programs in the variant discovery pipeline have different performance on plant genomic dataset. Choice of the programs is subjected to the goal of the study and available resources. This study serves as an important guiding information for plant biologists utilizing next-generation sequencing data for diversity characterization and crop improvement.

**Electronic supplementary material:**

The online version of this article (10.1186/s12864-019-6057-7) contains supplementary material, which is available to authorized users.

## Background

Genomic technologies provide unprecedented opportunities to reveal the history of crop domestication, to discover novel genetic diversity, and to understand the genetic basis of economically important traits, collectively contributing to crop improvement and food security [1]. One of the most important steps in genomic analyses is the ability to accurately and comprehensively identify genetic variations. As sequencing cost continues to decrease, whole genome sequencing (WGS) strategies are increasingly employed for plant diversity and domestication studies [2–5]. Accompanying improvements in sequencing technology is the need to not only improve but also better understand the algorithms that enable variant calling from sequencing data. Many of the algorithms used in the processing of sequencing data were originally developed and evaluated in human WGS studies yet are frequently used by plant genomic researchers [6–9]. The underlying assumption is that the performance of a given algorithm for human data will be similar for plant data, in spite of significant differences between the human and plant genomes.

The variant discovery pipeline for WGS dataset can be roughly divided into four steps: read mapping, variant calling, variant filtering, and imputation. Sequence aligners for the read mapping step can be grouped according to their indexing methodologies [9]. Programs such as Novoalign (http://www.novocraft.com) and GSNAP [10] use hash tables indexing methods; whereas BWA [11], SOAP2 [12] and Bowtie2 [13] use Burrows-Wheeler Transformation indexing algorithms. Variant calling programs can be categorized into alignment-based programs such as SAMtools [14] and FreeBayes [15], and assembly-based programs, such as GATK HaplotypeCaller [16] and FermiKit [17]. Variant filtering steps remove low-quality variants based on various quality metrics such as base quality, read depth, and mapping quality. The purpose of this step is to remove false positive variants while minimizing false negative variants, a source of “hidden diversity”. The basic filtering strategy, termed “hard-filtering” [18], sets empirical cutoffs on quality metrics to eliminate false positive variants.

Over the past decade, extensive progress in human genomic studies has developed and applied machine-learning based variant filtering methods [16] which uses adaptive cutoffs that adapt to a specific dataset, often by finding variants within the dataset that were previously identified with high confidence. The final step in variant discovery often employs imputation methods by leveraging external information to infer missing genotypes due to technical limitations. The standard way of imputation in human genomic studies utilizes a reference panel [19, 20], where a previously identified set of haplotypes link missing variants with successfully genotyped variants. Many of these advanced methods have yet to be readily adopted by plant researchers. In some instances, there are clear obstacles to implementation, such as the lack of extensive plant haplotype panels of similar quality to the 1000 Genomes Project [21] or HapMap [22]. Though some species, such as maize [23] and rice [24], are rapidly building these resources. Even though both plants and human genomics share a similar computational workflow, the structure and composition of plant genomes pose unique challenges that are not present in humans. As a result, the evaluation of these emerging computational genomic technologies is urgently needed in agriculture.

A major challenge for crop genomics is the ability to accurately and comprehensively characterize genetic diversity in domesticated crops, diverse landraces, and wild crop relatives. Genetic diversity in plants can be much greater than that found in human genomes. These sources of diversity, especially in the wild species, provide a reservoir of genetic variation for future crop improvement [25–27]. For example, introgression from related wild species into domesticated tomatoes has been used to improve agronomic performance such as abiotic tolerance [28–31]. For example, a gene from a wild relative of bread wheat has been shown to confer resistance to one of the most destructive stem rust pathogen races, Ug99 [32]. Characterizing these rich pools of diversity is an important challenge facing plant genomics because the regions containing this diversity may pose the most challenges for algorithms designed and optimized for human studies.

The second challenge for variant discovery in plant genomics is the quality of available reference genomes. The human reference genome has been in a constant state of improvement for decades (https://www.ncbi.nlm.nih.gov/grc/human). Once released, however, most plant reference genomes see little improvement, resulting in references that are less accurate and less complete than that found in humans. Other key challenges are the large amounts of repetitive sequences, structural variations and, in some crops, complex polyploid genomes [33, 34]. Diversity may be underestimated because of presence-absence variations (PAV) that are common to most plant genomes [35]. The diverse nature of plant genomes together with low quality or incomplete reference assemblies can negatively affect read alignment and variant calling steps, leading to inaccurate genotypes and missing variants [1, 36, 37].

Here, we benchmarked the performance of programs that are commonly used for variant discovery in plant studies. The comparison included three highly-cited sequence aligners, BWA-MEM, Bowtie2 and SOAP2, and two popular variant callers, GATK HaplotypeCaller (GATK-HC) and SAMtools mpileup (SAMtools-mpileup) using domesticated tomatoes, wild relatives and simulated genomic datasets. We show that as diversity and genome complexity increased, the ability of these algorithms to identify variants varied strongly. In addition, the inadequacy of a single reference genome was uncovered after a cross-reference comparison was performed. Finally, we evaluated the performance of machine learning based variant filtering method and reference panel assisted imputation methods on the high diversity plant datasets.

## Results

### Alignment program evaluation

The performance of three different aligners, BWA-MEM, Bowtie2, and SOAP2, was evaluated using Illumina paired-end read datasets from 52 domesticated tomato, 30 related wild relatives (Additional file 1: Table S1) [38], and simulated genomic sequences from different crops. Mapping percentage, alignment accuracy, and processing time for each aligner were evaluated.

**Fig. 1:**
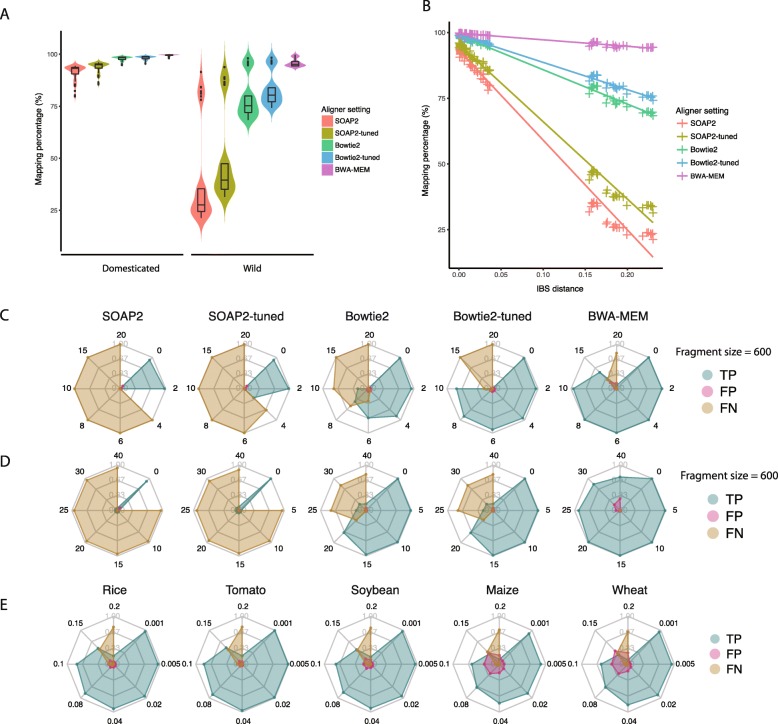
Aligner performance comparison using real and simulated plant genomic datasets. **a** Alignment percentage of five different aligner settings: SOAP2, SOAP2-tuned, Bowtie2, Bowtie2-tuned and BWA-MEM calculated for domesticated tomatoes and wild relatives. The width of violin plot is proportional to the density of the data. Boxplots inside violin plot indicate quantiles and outliers. **b** Mapping percentage of each sample is shown relative to the IBS distance to the reference genome. **c** Alignment accuracy of five aligner settings using simulated dataset with varied number of SNPs per read and fixed 600 nt fragment size. Each axis represents the number of SNPs in the corresponding simulation. The blue color represents percentage of true positive (TP) alignments, pink color represents the percentage of false positive alignment (FP) and gold color represents the percentage of false negative (FN) alignments. **d** Alignment accuracy of five aligner settings using simulated dataset with varied size of INDELs per read and fixed 600 nt fragment size. Each axis represents the size of INDELs in the corresponding simulation. **e** Alignment accuracy of BWA-MEM on different crop species. Each axis represents different mutation rate which includes both SNP and INDEL mutations

The ability to align reads to a domesticated tomato reference genome, *Solanum lycopersicum* [39], was assessed using default and tuned parameters on Bowtie2 (Bowtie2 and Bowtie2-tuned), SOAP2 (SOAP2 and SOAP2-tuned), and default parameters for BWA-MEM. Parameter tuning (see details in Methods) for Bowtie2 and SOAP2 was necessary to attempt to match the mapping percentage to the default used by BWA-MEM. BWA-MEM showed the highest alignment percentage, 99.54 and 95.95% in domesticated and wild relatives, respectively, while SOAP2 showed the lowest alignment percentage, 91.25 and 40.58%, respectively (Additional file 2: Table S2). In the domesticated tomato datasets, all of the five alignment settings resulted in more than 90% mapping percentage with standard deviation ranging from 0.34 to 3.77% (Fig. 1a). Greater variation in mapping percentage existed when analyzing the sequences from wild species with standard deviation ranging from 1.91 to 24.25%. The mapping percentage in the wild tomato samples displayed a bimodal distribution (Fig. 1a). The distribution of the group with higher alignment percentage contained wild species that were closely related to domesticated tomatoes, whereas the lower group contained distantly related wild species based on previous domestication and diversity studies [3, 40]. Alignment percentage was found to be negatively correlated with the IBS distance of each sample to the *S. lycopersicum* reference genome (Fig. 1b). When the sample was distantly related to the reference genome, BWA-MEM resulted in the highest mapping percentage and SOAP2 resulted in the lowest mapping percentage. In terms of processing time, SOAP2 was the fastest aligner in both domesticated and wild tomato datasets, and it was up to five times faster than the slowest alignment setting, Bowtie2-tuned (Additional file 6; Figure S1A).

We next determined whether greater alignment percentage or shorter alignment time could result in tradeoffs on accuracy and sensitivity by using simulated datasets and calculating the ratio of true positive (TP), false positive (FP) and false negative (FN) alignments. Simulated datasets were derived from the reference genome by permuting fragment sizes, and number of SNPs or size of small indels per read. For all alignment methods, the ratio of FP alignment increased as more SNPs or indels were introduced per read (Fig. 1c-d) when the fragment size was fixed at 600 nt. When the number of introduced SNPs was equal or less than 2, the average percent of FP alignments of BWA-MEM, Bowtie2-tuned and SOAP2-tuned was 0.94, 1.15 and 0.88%, respectively (Fig. 1c). When the number of introduced SNPs was greater or equal to 4, the average FP alignment rate of BWA-MEM, Bowtie2-tuned increased to 6.41 and 2.54%, respectively, while SOAP2, and SOAP2-tuned were no longer able to find alignments. BWA-MEM was the only aligner that was capable of finding TP alignments with 15 SNPs per read with FP alignment rate of 18.26%. Similar results were also observed in the indel simulation experiment (Fig. 1d). Only BWA-MEM was able to find TP alignments of reads with INDELs up to 40 nt in size at the cost of 26% false alignment rate. While differences in alignment percentages were observed, alignment length distributions were not found to differ for each aligner (Additional file 6: Figure S1B).

To indirectly determine the true vs false positive rates of BWA-MEM and Bowtie2 in real data, one million randomly selected reads from six samples (2 *S. lycopersicum*, 2 *S. pennellii* and 2 other wild relatives) were aligned to both *S. lycopersicum* and *Solanum pennellii* reference genomes [41]. The positions of alignments with mapping quality (MQ) ≥40 were compared against the synteny map of the genome generated by nucmer [42]. When the alignment position of read matched to the nucmer conversion of the *S. lycopersicum* coordinate to the *S. pennellii* coordinate, the read was considered to be syntenic. If the positions did not match, the read was considered non-syntenic. BWA-MEM was able to align approximately 4.22 times more reads per sample than Bowtie2 (Additional file 3: Table S3), but only 65.71% (*SD* ± 2.68%) of these alignments were considered as syntenic compared to 88.17% (*SD* ± 1.59%) of Bowtie2 alignments.

To extend the study to other crop species, simulated sequencing datasets were generated from rice, soybean, maize and wheat reference genomes by varying the mutation rate from 0.001 to 0.2 (Fig. 1e). In these studies, both SNP and INDEL were included in the simulation. When the mutation rate is equal to or lower than 0.04, BWA-MEM was able to align at least 92% of the sequences correctly for rice, tomato and soybean, whereas it was only able to correctly align 81.5 and 82% of the sequences for maize and wheat, respectively. As mutation rate increased, difference in both true positive and false positive alignment was seen among different crops. On average, BWA-MEM was able to find 18.1, 20.2 and 17.0% more true positive alignments in rice, tomato and soybean than in wheat and maize at mutation rate 0.08, 0.1, and 0.15, respectively. On the other hand, BWA-MEM was able to generate 18.8, 22.5, and 24.5% less false positive alignments in rice, tomato and soybean than in wheat and maize at mutation rate 0.08, 0.1, and 0.15, respectively.

### Variant calling program comparison

Four variant datasets were produced from the permutation of the aligners, Bowtie2-tuned, and BWA-MEM, and the variant callers SAMtools-mpileup and GATK-HC using 52 domesticated and 30 wild tomatoes. Results showed nearly a two-fold difference in the number of unfiltered SNPs ranging from 69.2 M to 133.7 M. A greater difference in the variant count in wild species was observed than that found in domesticated ones (Table 1). In domesticated species, dataset sizes ranged from 11.8 M to 17.8 M unfiltered SNPs, while in wild species they ranged from 66.4 M to 128.3 M. The primary determinant of variant count between datasets was whether Bowtie-2 or BWA-MEM was used. In domesticated species, 10.7 M SNPs were commonly identified by different aligners and variant callers, and when BWA-MEM was used as the aligner, about 83% (14.7 M) SNPs were identified by both GATK-HC and SAMtools-mpileup (Additional file 6: Figure S2A). In wild species, 59 M SNPs were commonly identified by different aligners and variant callers, and when BWA-MEM was used as the aligners, about 84% (109.8 M) SNPs were identified by both GATK-HC and SAMtools-mpileup (Additional file 6: Figure S2B). The inbreeding coefficient was calculated for each tomato individual, no significant difference (Wilcoxon rank sum test, *p*-value 0.47) was found between GATK-HC and SAMtools-mpileup identified SNP variants.

**Table 1:** Summary of SNPs identified by combinations of aligners and variant calling programs

	Unfiltered SNPs				Filtered SNPs			
	Total	Domesticated tomatoes	Wild tomatoes	Common	Total	Domesticated tomatoes	Wild tomatoes	Common
BWA-MEM + GATK-HC	131,449,946	17,771,072	128,294,973	14,616,099	93,739,759	13,628,974	91,482,115	11,371,330
Bowtie2-tuned + GATK-HC	73,393,338	11,813,500	70,453,383	8,873,545	30,307,811	8,261,729	28,243,136	6,197,054
BWA-MEM + SAMtools-mpileup	133,734,683	17,268,821	130,886,221	14,420,359	80,709,232	10,366,835	78,727,565	8,385,168
Bowtie2-tuned + SAMtools-mpileup	69,219,499	12,390,916	66,416,422	9,587,839	46,436,709	8,832,598	44,626,459	7,022,348

To further evaluate the differences in the ability of identifying variants, both individual-level and population-level simulated datasets were generated with varied mutation rates, sequencing coverage and population size. In the simulated population-level datasets, evaluation was performed on both raw and filtered variants. In the comparison of raw variants, GATK-HC was able to identify more true SNPs at the cost of accuracy as sequencing coverage increased in diversity populations. At 5x and 10x coverages, SAMtools-mpileup was able to identify similar recall ratio with higher precision ratio than GATK in the low diversity population. When dealing with high diversity populations, GATK-HC always outperformed SAMtools-mpileup in both precision and recall aspects (Additional file 6: Figure S2C). When functional annotation was applied to each identified coding SNP, nearly identical percentages of missense, nonsense and silent SNPs were found between GATK-HC and SAMtools-mpileup (Additional file 4: Table S4). In the comparison of raw INDELs, GATK-HC always outperformed SAMtools-mpileup in terms of precision and recall in the low diversity population. In the high diversity populations, GATK-HC was able to identify greater number of true INDELs at the cost of accuracy (Additional file 6: Figure S2D). The true size of simulated INDELs ranged from − 6 bp to 6 bp. The size of the raw INDELs identified by GATK ranged from − 170 bp to 241 bp, and size of the raw INDELs identified by SAMtools-mpileup ranged from − 5 bp to 7 bp.

**Fig. 2:**
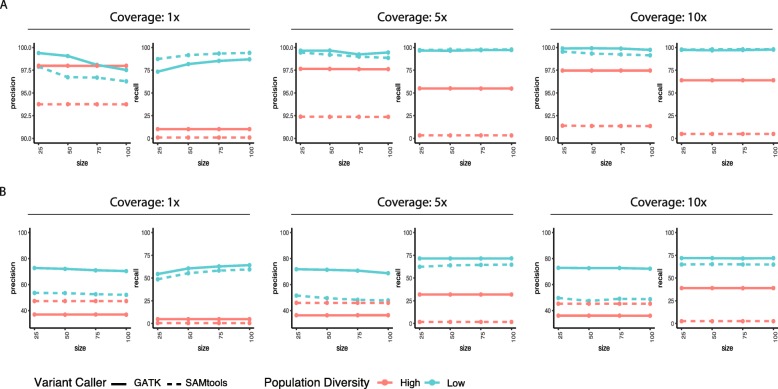
Evaluation of variant calling programs using simulated plant genomic datasets. **a** The comparison of the performance of GATK-HC and SAMtools-mpileup on filtered SNPs at different coverages, population diversity and population size. **b** The comparison of the performance of GATK-HC and SAMtools-mpileup on filtered INDELs at different coverages, population diversity and population size

In the filtered SNP results, when the sequencing coverage is at 5x and 10x, GATK-HC provided a higher precision ratio in all coverage and diversity permutations without compensating the recall ratio (Fig. 2a). In the 1x coverage simulation dataset, even though SAMtools-mpileup identified variants with lower precision ratio, it generated a higher recall ratio in the dataset. In the filtered INDEL results, GATK-HC always outperformed SAMtools-mpileup in terms of precision and recall ratio in the low diversity population. In the high diversity population, SAMtools-mpileup resulted in a higher precision ratio at the cost of a much lower recall ratio (Fig. 2b). Noticeably, SAMtools-mpileup was only able to result in 3.08 and 1.61% recall ratio in the high diversity populations for SNPs and INDELs, respectively.

In the individual-level simulated dataset, a consistent pattern of trade-off between precision and recall was observed. SAMtools-mpileup was able to generate higher precision ratio for both SNPs and INDELs, however, GATK-HC was able to result in a higher recall ratio for both SNPs and INDELs as coverage and mutation rate increased in most case (Additional file 6: Figure S3A-D). Among four different crop species, rice, tomato and soybean had similar results in both variant calling programs. Nevertheless, results from simulated maize datasets showed lower precision and recall ratios. Noticeably, when the mutation rate was at 0.1 and 0.15, both variant calling programs resulted lower precision ratio for SNP detection as coverage increased. Maize datasets had the largest magnitude of reduction in precision whereas other crop species resulted similar reduction.

### Wild reference genome alignment and variant calling

The large increase in the number of SNPs in wild samples was expected due to both greater distance from the domesticated reference genome and increased diversity relative to the domesticated samples. Expectedly, as the distance from the reference genome increased, a greater proportion of reads was unmapped. The variants in these unmapped reads, especially in the wild species, could represent “missing diversity”. To test this hypothesis, we evaluated how variants discovery in these 82 tomato samples were changed by mapping reads to a wild reference genome (*S. pennellii*) [41].

**Fig. 3:**
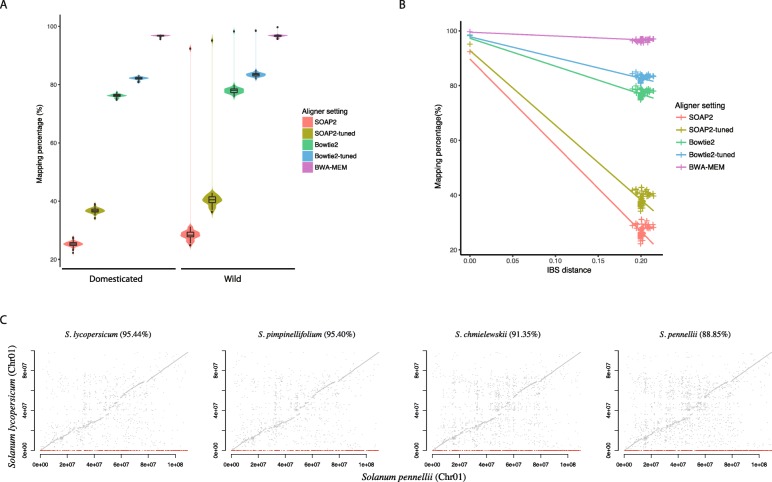
Alignment and variant calling using a wild reference *S. pennellii* genome. **a** Alignment percentage of five different aligner settings: SOAP2, SOAP2-tuned, Bowtie2, Bowtie2-tuned and BWA-MEM calculated for domesticated tomatoes and wild relatives using the *S. pennellii* reference genome. The width of violin plot is proportional to the density of the data, and boxplot is plotted inside violin plot showing quantiles and outliers. **b** Mapping percentage of samples for different aligner setting. The mapping percentages are relative to the IBS distance to the reference genome. **c** SNP identification of four tomato samples was performed in chromosome 1 in *S. pennellii* reference genome. The corresponding physical positions of SNPs in the *S. lycopersicum* reference were plotted. The grey dots represented the SNPs that were able to be located at the corresponding positions in *S. lycopersicum* genome, red dots represented the SNPs that were unable to be located to corresponding positions in *S. lycopersicum* genome. The percentage of corresponding SNPs were written next to the species name

The read alignment to the *S. pennellii* reference was performed under identical settings as above. As previously seen, BWA-MEM showed the highest mapping percentage and SOAP2 showed the lowest (Fig. 3a). In general, mapping percentage in domesticated and wild tomato groups were similar regardless of aligner settings used (Fig. 3a). The single outlier with high alignment percentage was a *S. pennellii* sample with an alignment of 95.13% (or 99.69%) as opposed to 34.22% (or 94.87%) against the *S. lycopersicum* reference using SOAP2-tuned (or BWA-MEM). Interestingly, the 82 samples, except for the *S. pennellii* sample, had similar IBS distances to the reference genome. As with the *S. lycopersicum* reference, alignment percentage to the *S. pennellii* reference was inversely proportional to IBS distance to the reference genome (Fig. 3b), suggesting this relationship was independent of reference genome used.

To investigate how diversity estimation varied by reference genome, reads from randomly selected eight domesticated tomatoes and eight wild relative accessions were aligned to the *S. pennellii* reference. Alignment to the *S. pennellii* reference genome generated a total of 96,712,749 unfiltered SNPs and 59,944,499 filtered SNPs, while a total of 77,718,102 raw SNPs and 53,036,666 filtered SNPs were identified using the *S. lycopersicum* reference genome. Compared to using the *S. lycopersicum* reference genome, significantly more SNPs (Two-sample T-test, *p*-value = 2.3*10^− 10^) were identified from 8 domesticated tomato samples when *S. pennellii* reference genome was used for variant discovery (Additional file 6 Figure S4A).

To further investigate the source of this additional variation, a cross-reference comparison was performed between SNPs identified using *S. pennellii* and *S. lycopersicum* reference genomes. One hundred nucleotides of DNA sequence flanking each filtered SNP identified using one reference genome was aligned to the other reference. Results in the Fig. 3c showed that majority of the filtered SNPs identified in the *S. pennellii* located on the synteny path of *S. lycopersicum* genome. In the *S. lycopersicum* sample, and similarly, majority filtered SNPs identified using *S. pennellii* reference were located on the synteny path of *S. lycopersicum* genome. This result indicated that using *S. pennellii* reference genome, we were able to identify SNPs that were fixed in the *S. lycopersicum* domesticated varieties.

Since these SNPs were fixed in *S. lycopersicum*, they would not have been identified from alignment to the *S. lycopersicum* reference. Outside of these fixed SNPs in the domesticated species, 4.55% of flanking sequences of SNPs identified using *S. pennellii* genome in chromosome 1 could not be mapped to the *S. lycopersicum* reference. Similarly, 11.15% of the flanking sequences of SNPs identified in the *S. pennellii* sample using the *S. pennellii* genome were not found in the *S. lycopersicum* genome (Fig. 3c). Switching to the domesticated reference genome, 7.13% of the downstream sequences of SNPs identified in a *S. lycopersicum* sample using *S. lycopersicum* genome could not be found in the *S. pennellii* genome (Additional file 6: Figure S4B). These results indicated that a great portion of variation in the wild species would be missed if a single domesticated genome was used as the reference, and vice versa.

### Hard-filtering and machine-learning based variant filtering

Variant filtering is required to minimize both false positive and negative genotype calls. Comparisons were made between three variant filtering methods: setting empirical hard-cutoffs (HARD) on metrics such as read depth, strand bias, and variant quality and so on, a newly implemented machine-learning based (ML) variant filtering [16], and a combination between HARD and ML (COMBINED) filtering. Filtered datasets generated from the 602 WGS tomato datasets, including a wide range of domesticated and wild tomato samples [28], were analyzed. A training dataset of 8401 markers from SolCap was used for the training phase of ML [43]. The SolCap is a high confidence dataset consisting of verified markers previously used in genetic studies. In the COMBINED method, the HARD filters were first applied to SolCap to remove low-confidence markers and yield a training set of 7633 variants. Results indicated that the HARD-filtered method retained the fewest SNPs (94.2 M), which was 26.3 and 7.1% fewer than ML-filtered (127.8 M) and COMBINED-filtered (101.4 M) datasets, respectively (Additional file 5 Table S5). SNPs in the first 10 million bases in Chromosome 1 (Additional file 5 Table S5) were cross-compared between the three datasets. 70% of SNPs in this segment were shared among all three filtered datasets (Additional file 6 Figure S5A), while each dataset had a subset of unique variants.

**Fig. 4:**
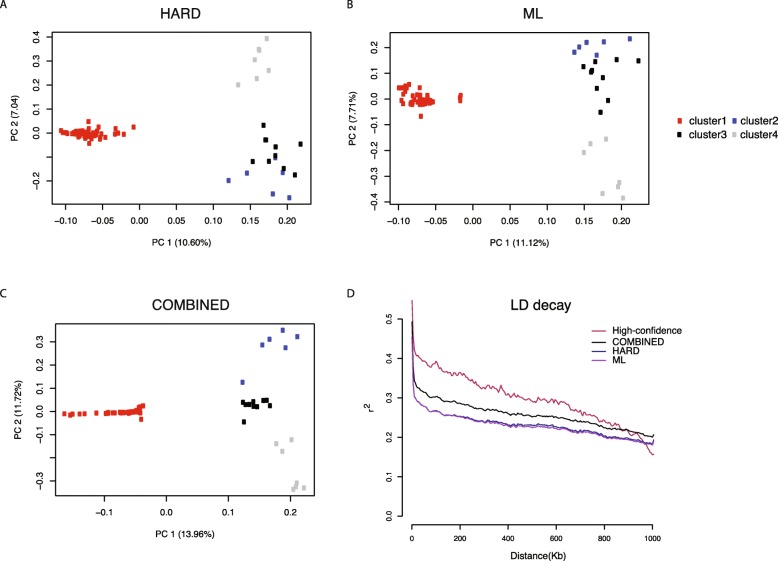
Comparison between three variant filtering methods using PCA and LD decay to estimate false positive and false negative ratios. **a** Unshared HARD filtered SNPs were not able to clearly separate cluster 2 and 3. **b** Unshared ML filtered SNPs were not able to clearly separate cluster 2 and 3. **c** Unshared COMBINED filtered SNPs were able to clearly separate 4 clusters. **d** Comparison of LD decay among four sets of SNPs

Two methods were used to indirectly infer the quality of filtered datasets: recapitulation of diversity estimates generated by a “gold standard” set of 22,336,965 SNPs (See details in Methods) in the form of PCA (Additional file 6 Figure S5B) and IBS analyses (Additional file 6; Figure S5C), and calculation of LD decay distance for each filtered dataset. SNPs identified by all three filtering methods were removed for this analysis so that the efficacy of each method could be evaluated independently. The underlying assumption of these analyses was that true diversity would recapitulate the known population structure, whereas the population structure would begin to break down as the number of artifacts increased. Using the “gold standard” variant dataset, samples were grouped into four clusters based on PCA and IBS results. All three filtering methods were able to resolve Cluster 1 and Cluster 4, whereas the HARD and ML filtering methods were not able to clearly resolve Cluster 2 from Cluster 3 (Fig. 4a-b). In contrast, the COMBINED filtering method was able to identify all four original clusters to reconstruct the population structure of 82 Solanum genomes (Fig. 4c).

Next, the contribution of false positive SNPs in each filtered dataset was evaluated by calculating the rate of LD decay. The assumption was that false positive SNPs were random noise that would be found not in LD with nearby SNPs. Therefore, the apparent rate of LD decay in a dataset would increase as the number of false positives increased. As predicted, a greater rate of LD decay was found in all three filtered datasets than that found in the high-confidence dataset. Of the three filtered datasets, the COMBINED method, however, had the lowest rate of LD decay (Fig. 4d) approximating the rate of LD decay seen in the high-confidence SNP dataset.

To quantitively measure the difference between hard filtering and machine-learning based filtering, simulated datasets with varied population size, mutation rate and sequencing coverage were generated (Additional file 6: Figure S6A-B). In the simulation analysis, 30% of the simulated gold standard variants were used as the training dataset, and no hard-filtering was performed on the training dataset. In the low diversity population datasets, machine-learning based SNP filtering always outperformed hard SNP filtering by 7.38 and 14.14% on average for precision and recall ratio, respectively. In terms of INDEL filtering in the low diversity dataset, machine learning based filtering and hard filtering resulted comparable precision results, however, machine learning based filtering was able to result 12.49% higher recall ratio than hard filtering. In the high diversity population, SNP and INDEL had similar results from different filtering methods. Minor difference was observed in the recall ratio between machine-learning based and hard filtering. No difference was found in the precision ratio between machine-learning based and hard filtering in the high diversity population.

### Two-step imputation method

Missing genotypes, possibly due technical limitations, are commonly resolved via imputation. In human studies, standard imputation methods leverage linkage disequilibrium (LD) and reference panels [44]. Beagle 4.1 is a commonly used imputation algorithm in plant studies that can function with or without a reference panel. To determine the importance of a reference panel for SNP imputation, both LD-based and reference panel-assisted imputation were applied to several datasets. A reference panel of 22,336,965 high-confidence, phased SNPs was generated from 82 high coverage (30x) WGS tomato datasets. Imputation results were compared between the two methods. In the first method, missing SNPs were imputed without a reference panel. In the second method, imputation was performed in two steps: in the first step a reference panel was used to impute missing calls only for missing reference variants; and then a second step was employed to impute the remaining missing, non-reference SNPs. Samples were placed in four groups and varying percentages of high confidence genotypes were masked to act as “missing” data (See details in Methods). The concordance (*r*-squared) between the original masked and imputed genotypes was calculated to estimate imputation accuracy.

**Fig. 5:**
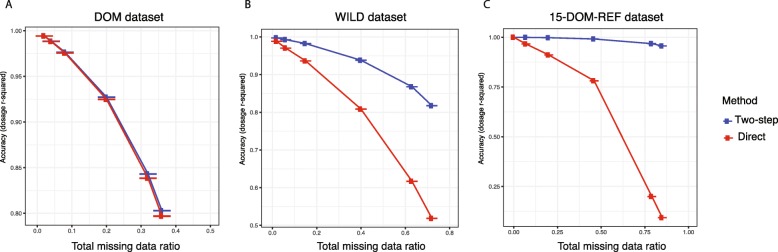
Comparison of imputation accuracy using direct imputation and 2-step imputation methods. **a** Imputation accuracy using direct imputation and 2-step imputation relative to missing SNPs in 100 domesticated tomato samples. **b** Imputation accuracy using direct imputation and 2-step imputation relative to missing SNPs in 36 wild samples. **c** Imputation accuracy using direct imputation and 2-step imputation relative to missing SNPs in 15 domesticated samples used in reference panel

Results showed that no difference between LD-based and 2-step imputation was observed in 100 domesticated (DOM) tomato samples (Fig. 5a) or the 50 *Solanum pimpinellifolium* (PIM) samples (Additional file 6: Figure S7A) datasets. In the dataset of 200 randomly selected tomato samples (RANDOM), at 47% missing data, a 4% difference was observed (Additional file 6: Figure S7B). When the parameter of missing percentage was set at 72%, 2-step imputation methods showed 60% higher accuracy than LD-based imputation in the dataset of 36 wild tomato species (WILD) (Fig. 5b). High LD between SNPs may reduce the need for a reference panel in imputation. The calculated LD decay for each dataset showed that DOM had the slowest LD decay and WILD had the fastest LD decay (Additional file 6: Figure S7C). Due to the fact that limited samples of wild tomato were available, the number of samples we used in the simulation in DOM (100) was also considerably higher than that in WILD (36). As such, considerably more information was present in the DOM dataset for imputation, as opposed to the WILD dataset which not only had a smaller number of samples but also contained multiple species. To determine if LD continued to be sufficient for imputation in small domesticated panels when the amount of missing data was considerable, 15 randomly selected domesticated tomato samples that were also included in the reference panel (15-DOM-REF) had up to 85% of their genotypes masked. Both methods were applied to the 15-DOM-REF dataset. The results showed two-step imputation was 9.25 times more accurate than Beagle v4.1 direct imputation by when the missing percentage was 85% (Fig. 5c).

## Discussion

The ability to accurately and comprehensively identify genetic variation is a critical step for studying diversity, trait mapping and breeding in plant genomics. Many plant studies involve high levels of genetic diversity and, in some instances, incorporating distantly related varieties and wild relatives. Neither of these conditions are common in human studies, and as such pipelines designed and evaluated on humans may perform differently than expected. Therefore, we evaluated programs that are commonly used by plant genomic studies on SNP discovery steps including read alignment, variant calling, variant filtering and missing data imputation in the context of plant diversity discovery.

One of the first computational steps in the variant discovery pipeline is the alignment of reads to a suitable reference genome. Previous aligner evaluation studies have been performed using either human or microbial genomic datasets [45, 46], which may not represent the levels or types of diversity expected in plant studies. We performed alignment using both real and simulated plant datasets and found that different aligners were very different in their tolerance of sequence variation in paired-end reads. BWA-MEM outperformed four other alignment settings in mapping percentage while still being able to maintain high mapping accuracy. Neither SOAP2 nor Bowtie2 was able to align as many reads, even after optimizing their settings to account for increased variation. In this study, we chose not to tuned BWA-MEM mostly because the mapping percentage was high with the default settings and there is no obvious parameter such as numbers of mismatches allowed, or fragment size as found in Bowtie2 or SOAP2. Besides, many program users, especially non-experts in bioinformatics, may stay with the default settings of programs.

BWA-MEM’s increased sensitivity may come at a cost in that, as the number of SNPs or size of INDELs per read increased, the false positive rate also became slightly higher than that of Bowtie2-tuned (Fig. 1c-d). The increased number of false positive alignments may, in turn, result in erroneous variant identifications. Nevertheless, given the relatively high sensitivity and accuracy of BWA-MEM, our results indicate that under most circumstances it is probably the most suitable algorithm for read mapping for plant datasets, especially when distantly related samples are included in the analysis. If high accuracy at the cost of less sensitivity is desired, Bowtie2 may be the better choice. Although SOAP2 was the fastest aligner tested, its difficulty in aligning reads with high variance from the reference genome make it unsuitable for studies where significant levels of genomic diversity may be present.

The next step in an analysis pipeline is variant calling. Comparisons between aligner-variant caller combinations indicated that the alignment algorithm had a greater impact on the number of variants discovered than the variant caller used. For a given aligner, SAMtools-mpileup and GATK-HC had similar results in the total number of SNP identified in the real tomato genomic dataset. This further emphasizes the importance of selecting an aligner appropriate to the goals of the experiment, especially when high diversity samples such as wild relatives and related species are included in the study. According to the simulation results, GATK-HC was able to identify more true positive variants at higher precision ratio in most population-based variant discovery cases. Especially in the high diversity population simulation, GATK-HC was more preferred than SAMtools-mpileup because SAMtools-mpileup resulted very low recall ratio in both SNP and INDEL detection. In the simulation analysis, the size of INDELs identified by GATK-HC had larger range than those identified by SAMtools-mpileup and ground truth INDELs, which partially explained why GATK-HC had lower precision in the INDEL identification than SAMtools-mpileup. One of the possible explanations is that GATK-HC performs local-assembly to identify the haplotypes whereas SAMtools-mpileup only utilizes read alignments. Plant genomes, in general, are rich in repetitive sequences which are difficult to assemble correctly using short reads. Therefore, the local assembly strategy taken by GATK-HC will not only identify true variants, but also generate false positive variants, INDELs especially. Consistent with a previous research [47], SAMtools-mpileup resulted higher precision value than GATK-HC for variant identification in the individual-based genotyping. However, the high precision of SAMtools-mpileup is at the trade-off of low recall value.

In general, we recommend GATK-HC for variant calling and filtering for several reasons. First of all, GATK-HC outperformed SAMtools-mpileup in most of our situation tests resulting a higher precision and recall ratio for SNP and INDEL detection. Second, GATK-HC allows rapid incorporation of multiple samples into a dataset without needing to recall genotypes for all samples, even previously genotyped ones, from aligned reads by using the GVCF system. This saves considerable time and computational expense when adding samples to a dataset. The third reason to recommend GATK-HC is that it supports multi-thread processing which is not available in the SAMtools-mpileup. Taking the advantage of high-performance clusters, multi-thread feature can significantly save processing time especially for large studies. Finally, the GATK package supports sophisticated machine learning based variant filtering (VQSR) which showed superior performance than empirical hard cutoffs. We did, however, find situations that SAMtools-mpileup is more preferable depending on the goal of the study. For example, for a low diversity population with very low sequencing coverage (1x), SAMtools-mpileup was able to identify more true SNPs than GATK-HC but at the cost of lower precision. If the purpose of the experiment is to identify as many true positive SNPs as possible, then SAMtools-mpileup could be used in this particular situation. Another situation that SAMtools-mpileup may be preferable is identifying SNPs from a closely related sample. According to the simulation results from single samples, SAMtools-mpileup resulted slightly higher precision and recall values than GATK-HC results when the mutation rate was lower than 0.05. If the experiment aims at charactering SNPs in a genotype that is closely related to the reference genome, SAMtools-mpileup could be used in this particular situation.

Variant filtering is the third step in a diversity assessment pipeline. Three approaches to this were evaluated: hard filtering of various quality metrics, machine learning as implemented in GATK, and a combined approach. The combined approach which utilized hard filtered SolCap markers as the training dataset showed significant improvements over other variant filtering methods. According to the PCA plots (Fig. 4a-c) and LD decay figure (Fig. 4d), the combined method was able to generate more true positives, with fewer false negative SNPs and fewer false positive SNPs when an appropriate training dataset was used. This indicates that machine-based learning methods may be better suited at identifying true positives and eliminating false positive SNPs than empirical hard-filtering. The difference in the results of combined and machine learning filtering suggested the importance of the training dataset. The machine learning model will learn from errors in the training dataset that might contribute to false positive variants. The downside of machine-learning-based filtering is that its implementation is complicated and requires experimentally validated (high-confidence) training datasets. In human studies, this information can be obtained from numerous genomic resources such as HapMap, the 1000 Genomes Project and omni SNP array datasets. Only in few major crops, such as maize [23], rice [48] and soybean [49] have these resources available. Similar conclusions were found from the simulation tests. According to the simulation results, machine learning filtering outperformed hard filtering in general. Nevertheless, only minor difference was found when the simulated population had high diversity for both SNP and INDEL filtering suggesting the quality metrics used by machine learning filtering may not be sophisticated enough to differentiate true variants from false positive variants. This also indicates new quality metrics may be necessary, especially for the genomic regions that can be hyper-variable.

The final step in the variant discovery pipeline is imputation. Reference panels are routinely employed in human studies, but these have not been routinely employed in plant genomics. To evaluate the importance of a reference panel for imputation, Beagle v4.1 [19] was used to impute masked genotypes in four sample groups without the use of a reference panel and with a reference panel in a two-step process where SNPs contained in a reference panel were first imputed, and then imputation was extended to the entire dataset. Our results showed that the two-step imputation method was able to utilize a de novo reference panel of SNPs generated from high coverage sequencing data to assist imputation in the low coverage samples. Results from these studies indicated that the two-step imputation method was superior to the LD-based imputation method in sample groups that contained wild species. In addition, even for closely related samples, a certain number of samples must be present for LD-based imputation to produce valid results. Further, if there are insufficient samples, a reference panel may be required (Fig. 5c). The tradeoff was that 2-step imputation doubled the running time and would incorrectly impute missing SNPs which were not due to technical issues but because of structural variations. Therefore, care must be taken not to introduce false positive since presence-absence variations are common in plants. These genomic regions could be identified prior to imputation to avoid this pitfall.

The effect of presence-absence variation on identifying missing genetic diversity is a special concern in studies that include high diversity samples. This issue can be seen from the results of the cross-reference experiment. Up to 11.15% of the variations identified using the wild reference could not be mapped back to the domesticated *S. lycopersicum* genome, and vice versa. These results indicated the inadequacy of single reference genome for comprehensive variant discovery. It also indicated that employing multiple reference genomes could identify additional sources of diversity that went undetected when using a single reference. These results have implications for the utility of pan-genomes. Multiple references or pan genomes would likely increase the detection of “missing diversity” that is due primarily to PAV between samples. Moreover, using a distantly related reference genome may allow the detection of SNPs that would be undetected using a closely related reference genome. These species-specific, fixed variants have implications in the evolutionary history of plant species such as domestication events. To date, several crop pan-genomes have been reported [50–52] that show significant amount of structural variations in the genome. Pan-genomes resources should be included into the diversity discovery pipeline in the future. Yet, one of the potential issues that will need to be addressed is that pan-genome assembled from diverse individuals may introduce more assembly errors than a single reference assembly. The quality of the reference genome will impact variant discovery because bioinformatic tools assume the reference genome is correct and only identify differences accordingly. Moreover, the level of heterozygosity of the reference introduced by the pangenome may require additional fine-tuned parameters [53]. The most effective approach of utilizing a pan-genome reference will be a subject of future investigation.

## Conclusion

In conclusion, we found that BWA-MEM was better overall at detecting more true-positive alignments, especially in distantly related samples, while Bowtie2 was better at minimizing the incorrect alignments. Incorporating multiple reference genomes gave a more complete picture of variations, especially when the samples showed considerable presence-absence variations. For filtering, the optimal approach found in our test was to incorporate a combination of machine learning and hard filtering, in which a set of “known” SNPs was used as the training set for machine learning. This requires a panel of known, high-quality SNPs, however, which may be unavailable for many plant species. Finally, the importance of high-quality reference panels was emphasized during the imputation step especially when genotype imputation was challenging due to small LD blocks or not enough samples. Above all, the computational pipeline to discover variation from plant sequencing data will depend upon the diversity of the datasets, whether the goals of the experiment benefit from higher sensitivity or accuracy, the depth of sequence coverage, and the availability of external resources such as reference panels and gold-standard SNPs.

## Methods

### Simulated multi-species genomic dataset and real tomato genomic dataset

We used publicly available 602 WGS datasets representing 514 domesticated and 88 related wild species of tomato. The data were retrieved from the NCBI BioProjects under accession PRJNA259308, PRJNA353161 and PRJEB5235. The raw sequence data was quality trimmed using Trimmomatic (version 0.36) [54] with the options ILLUMINACLIP:TruSeq3-PE-2.fa:2:30:10:8:TRUE SLIDINGWINDOW:4:20 LEADING:5 TRAILING:5 MINLEN:36. PCR duplicates were removed using Picard MarkDuplicates (version 2.14.1) (http://broadinstitute.github.io/picard/). Simulated tomato sequencing reads were generated from the *S. lycopersicum* reference genome. A custom Python script was used to introduce from 0 to 20 SNPs per read, fragment sizes ranging from 200 to 10,000 nt, and INDELs ranging from 0 to 40 nt. In order to evaluate the performance of BWA-MEM on multiple crop species, simulation of the Illumina sequencing reads was also performed on rice, soybean, tomato, maize and wheat using mason (version 2.0.9) [55]. The mutation rate including SNPs and INDELs was simulated at 0.001, 0.005, 0.02, 0.04, 0.08, 0.1, and 0.15. The proportion of the SNPs and INDELs were 0.85 and 0.15, respectively. Sequencing error was modeled as the default settings.

### Evaluation of read alignment programs

Different aligners were evaluated using high-coverage datasets from PRJEB5235 and simulated datasets. BWA-MEM (version 0.7.17-r1188), SOAP2 (version 2.21), SOAP2-tuned, Bowtie2 (version 2.3.3.1) and Bowtie2-tuned were tested. SOAP2-tuned was used with the following options: -m 100 -× 888 -s 35 -l 32 -v 3 [28]. Bowtie2-tuned was used with the following options: --very-sensitive -N 1 -I 100 -X 888. To determine mapping percentages, these five aligner settings were used to align one million reads that were randomly selected from high coverage genomes from 52 domesticated and 30 wild relative samples. The IBS (Identity-By-State) distance was calculated using SNPrelate (version 1.16.0) [56]. The true positive alignment ratio was calculated by comparing the known ground truth location and aligned location. BWA-MEM was also evaluated on multiple crop species with a mixture of SNPs and INDELs in the simulated datasets.

### SNP discovery comparison

Eighty-two high-coverage datasets from PRJEB5235 was used for SNP discovery comparisons. SNPs were called with SAMtools-mpileup (version 1.9) and GATK-HC (version 3.8–0-ge9d806836) using BWA-MEM and Bowtie2-tuned alignments. In GATK, variants were firstly identified by HaplotypeCaller using the option --emitRefConfidence GVCF, and then joint genotyping was performed using GenotypeGVCFs. In SAMtools-mpileup, genotyping was done in one step and the option -C 50 was used as recommended in the manual. Only polymorphic SNPs were used as data for the Venn diagram. Simulated datasets with known variants were generated for tomato, rice, soybean, maize using mason. Each crop species was simulated at different coverages (5x, 15x, 30x, and 50x) and mutation rates (0.001, 0.01, 0.05, 0.1, 0.15). In addition to individual simulated datasets, population-level simulated datasets were also generated with varied diversity (low diversity: 0.001 mutation rate and high diversity: 0.1 mutation rate), population size (25, 50, 75, and 100) and sequencing coverage (1x, 5x, and 10x). SAMtools-mpileup and GATK-HC were evaluated on both individual and population simulated datasets by comparing the precision and recall ratios. The functional annotations of the variants were predicted by snpEff (version 4.3) [57].

$$ Precision=\frac{True\ Positive}{True\ Positive+ False\ Positive} $$

$$ Recall=\frac{True\ Positive}{True\ Positive+ False\ Negative} $$

### Imputation algorithm comparison

Beagle v4.1 [19] direct imputation and 2-step imputation method were compared using 602 tomato genomes. The raw SNPs were called using BWA-MEM and GATK-HC pipeline, and then hard filtered using GATK recommended options: “QD <2.0 || FS > 60.0 || MQ < 40.0 || SOR > 3.0”. The high-confidence set of SNPs for the 2-step imputation was identified from 82 high-coverage dataset using BWA-MEM and GATK-HC. GATK hard-filtering and VCFtools [58] with options: --missing 1 and --mac 2. SNPs with heterozygosity above 20% were removed. Beagle v4.1 was used to phase the high-confidence set of SNPs. The comparison was performed on four groups of samples: 200 random tomato and wild samples (RANDOM), 100 domesticated tomato samples (DOM), 50 *Solanum pimpinellifolium* samples (PIM), and 36 distantly related wild species (WILD). The one hundred domesticated samples from PRJNA353161 only, 15 DOM-REF samples from PRJEB5235 only, 50 PIM samples and 36 WILD samples were randomly selected for generating simulated datasets. Polymorphic SNPs in each dataset were randomly masked using a custom Python script if there were more than 7 reads supporting the genotypes. Both Beagle v4.1 and 2-step imputation methods were used to impute missing genotypes in five simulated datasets. The concordance *R*^*2*^ ratio between genotyped and imputed values were calculated as imputation accuracy using BCFtools [59].

### Variant filtering algorithms comparison

The 602 tomato datasets were used to generate raw SNPs using BWA-MEM and GATK-HC pipeline. Hard-filtered, machine-learning based and combined filtering methods were individually applied to the raw dataset. The parameters used for hard-filtering included QualByDepth (QD < 2), FisherStrand (FS > 60), RMSMappingQuality (MQ < 40) and StrandOddsRatio (SOR > 3.0), which were suggested by the GATK hard filtering tutorial (https://gatkforums.broadinstitute.org/gatk/discussion/2806/howto-apply-hard-filters-to-a-call-set). For INDELs, hard filtering was performed using “QD <2 || FS > 200 || ReadPosRankSum < -20”, as suggested by the GATK tutorial. The machine learning based methods, for both SNPs and INDELs, followed the GATK Best Practice Workflow (https://software.broadinstitute.org/gatk/documentation/article.php?id=2805). To summarize, the first step was to build a variant recalibration model using the program VariantRecalibrator. In the real tomato genomic dataset, SolCap and filtered SolCap markers were used as the training dataset with prior likelihood set to 90 and 95%, respectively. In the simulated dataset, 30% of the simulated gold standard variants were used as the training dataset with the prior likelihood set to 95%. All the annotations generated by GATK-HC including coverage, coverage by depth, FisherStrand, StrandOddsRatio, MappingQualityRankSumTest, ReadPosRankSumTest, RMSMappingQuality and InbreedingCoeff, were used to build the recalibration model. The second step was to apply the recalibration model to variants using the program ApplyRecalibration with the option --ts_filter_level 99.9. Polymorphic SNPs in the first 10 million base pairs of Chromosome 1 were selected to test the performance of different filtering methods. PCA was performed using SNPrelate after LD pruning (R^2^ > 0.2). LD decay was calculated using the PopLDdecay package [60] with default parameters.

## Additional files

Additional file 1: **Table S1.** Summary of 82 tomato accession (XLSX 53 kb)

Additional file 2: **Table S2.** Summary of alignment time (XLSX 39 kb)

Additional file 3: **Table S3.** Summary of synteny analysis (XLSX 11 kb)

Additional file 4 **Table S4.** Functional annotation summary of variants identified by different variant calling programs (XLSX 9 kb)

Additional file 5: **Table S5.** Summary of different variant filtering results (XLSX 33 kb)

Additional file 6: **Figure S1.** Alignment time and length comparisons of different aligners. **Figure S2.** Evaluation of variant calling programs using real and simulated plant genomic datasets. **Figure S3.** Evaluation of different variant calling programs on simulated single genomic dataset. **Figure S4.** Cross-reference comparison on SNP identification. **Figure S5.** Machine-learning based variant filtering. **Figure S6.** Quantitative comparison between machine learning and hard-filtering using simulated dataset. **Figure S7.** Comparison between direct and two-step imputation (DOCX 27699 kb)

## Data Availability

The publicly available whole genome resequencing datasets were deposited in NCBI BioProjects under accession PRJNA259308, PRJNA353161 and PRJEB5235.

## Abbreviations

Bowtie2-tuned: Aligner Bowtie2 with tuned settings
DOM: 100 Domesticated tomato samples
FN: False negative
FP: False positive
GATK-HC: GATK HaplotypeCaller
PAV: Presence absence variation
PIM: 50 *Solanum pimpinellifolium* samples
RANDOM: 200 random tomato and wild samples
Soap2-tuned: Aligner Soap2 with tuned settings
TP: True positive
WGS: Whole-genome sequencing
WILD: 36 Distantly related wild species
