# Supplementary material for: Benchmarking variant identification tools for plant diversity discovery
Source: BMC Genomics. 2019 Sep 9;20:701. doi: 10.1186/s12864-019-6057-7 (PMC6734213; doi:10.1186/s12864-019-6057-7)
Supplement: Supplementary file 6 — Figure S1. Alignment time and length comparisons of different aligners. Figure S2. Evaluation of variant calling programs using real and simulated plant genomic datasets. Figure S3. Evaluation of different variant calling programs on simulated single genomic dataset. Figure S4. Cross-reference comparison on SNP identification. Figure S5. Machine-learning based variant filtering. Figure S6. Quantitative comparison between machine learning and hard-filtering using simulated dataset. Figure S7. Comparison between direct and two-step imputation (DOCX 27699 kb) [file 12864_2019_6057_MOESM6_ESM.docx]

**Supplemental Data**

**Supplemental Figure 1. Alignment time comparison of different aligners**

1. Alignment percentage of five different aligner settings: SOAP2, SOAP2-tuned, Bowtie2, Bowtie2-tuned and BWA-MEM calculated for domesticated tomatoes and wild relatives. The width of violin plot is proportional to the density of the data. Boxplots inside violin plot indicate quantiles and outliers.
2. The alignment length distribution of different aligners. Only alignment shorter than 96 nt was plotted for better visualization.


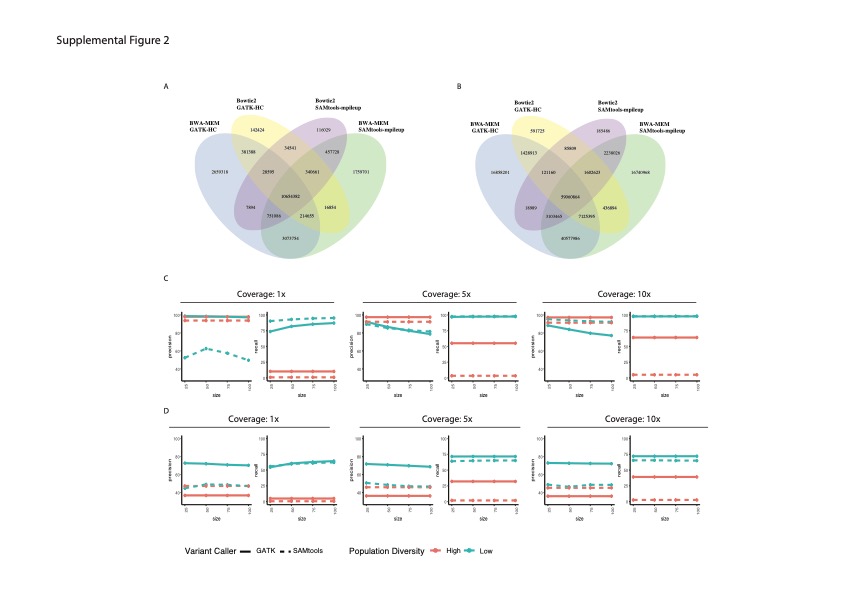


**Supplemental Figure 2. Evaluation of variant calling programs using simulated plant genomic datasets**

1. The Venn diagram of SNPs identified from domesticated tomato dataset using different aligner and variant caller combinations.
2. The Venn diagram of SNPs identified from wild tomato dataset using different aligner and variant caller combinations.
3. The comparison of the performance of GATK-HC and SAMtools-mpileup on raw SNPs at different coverages, population diversity and population size.
4. The comparison of the performance of GATK-HC and SAMtools-mpileup on raw INDELs at different coverages, population diversity and population size.

**Supplemental Figure 3. Evaluation of different variant calling programs on single genomic dataset**

1. SNP precision results of GATK-HC and SAMtools-mpileup on single simulated dataset with varied coverages, mutation rates and crop species
2. SNP recall results of GATK-HC and SAMtools-mpileup on single simulated dataset with varied coverages, mutation rates and crop species
3. INDEL precision results of GATK-HC and SAMtools- on single simulated dataset with varied coverages, mutation rate sand crop species
4. INDEL recall results of GATK-HC and SAMtools-mpileup on single simulated dataset with varied coverages, mutation rates and crop species

**Supplemental Figure 4. Cross-reference comparison on SNP identification**

1. Number of SNPs identified using *S. lycopersicum* or *S. pennellii* genome assembly as the reference
2. SNP identification of four tomato samples was performed in chromosome 1 in *S. lycopersicum* reference genome. The corresponding physical positions of SNPs in the *S. pennellii* reference was plotted. The grey dots represented the SNPs that were able to be located at the corresponding positions in *S. pennellii* genome, red dots represented the SNPs that were unable to be located to corresponding positions in *S. pennellii* genome. The percentage of corresponding SNPs are written next to the species name.

**Supplemental Figure 5. Machine-learning based variant filtering**

1. Venn diagram of SNPs in the 10M region of Chromosome 1 using HARD, ML and COMBINED filtering methods
2. Population structure of 82 tomato genomes using high-confidence SNPs
3. IBS distance of 82 tomato genomes using high-confidence SNPs

**Supplemental Figure 6. Comparison between VQSR and hard-filtering**

1. The comparison of the performance of VQSR and hard-filtering on SNPs at different coverages, population diversity and population size.
2. The comparison of the performance of VQSR and hard-filtering on INDELs at different coverages, population diversity and population size.

**Supplemental Figure 7. Comparison between direct and two-step imputation**

1. Imputation accuracy using direct imputation and 2-step imputation relative to missing SNPs in 200 random tomato samples
2. Imputation accuracy using direct imputation and 2-step imputation relative to missing SNPs in 50 *S. pimpinellifolium* tomato samples
3. Comparison of LD decay of SNPs from different populations.

Supplemental Table 1. Summary of 82 tomato accession

(See excel file SuppTable1_82accession_summary.xlsx)

Supplemental Table 2. Summary of alignment time

(See excel file SuppTable2_alignment_time_summary.xlsx)

Supplemental Table 3. Summary of synteny analysis

(See excel file SuppTable3_SyntenyAnalysisResults.xlsx)

Supplemental Table 4. Functional annotation summary of variants identified by different variant calling programs

(See excel file SuppTable4_functional_annotation.xlsx)

Supplemental Table 5. Summary of different variant filtering results

(See excel file SuppTable5_variant_filtering.xlsx)
